# Turkish adaptation, validity, and reliability study of the Quality of Life Gravidarum (QOL-GRAV) scale

**DOI:** 10.1186/s12884-025-07159-1

**Published:** 2025-02-04

**Authors:** Gamze Ayan, Serap Ejder Apay

**Affiliations:** 1 Bayburt State Hospital, Bayburt, Turkey; 2 Department of Midwifery, Ataturk University, Erzurum, Turkey

**Keywords:** Midwife, Pregnancy, Quality of life, Reliability, Validity

## Abstract

**Background:**

The study aims to adapt the validity and reliability of the Pregnancy Quality of Life Scale in Turkish society.

**Methods:**

The study was carried out methodologically between April 2021 and April 2022 at Bayburt State Hospital, the only public hospital operating in the city center of Bayburt. In the study, a total of 355 pregnant women who met the research criteria and volunteered to participate were examined without selecting a sample. Validity and reliability analysis of language and content validity, explanatory and confirmatory factor analysis, and Cronbach- α coefficient were used.

**Results:**

The factor structure of the Turkish form of the scale was consistent with the original form. As a result of the explanatory appropriate. The internal consistency coefficient was calculated as the total Cronbach-α coefficient of the scale was 0.628 for the first trimester, and 0.628 for the II. trimester 0.727 for trimester, III. Trimester it is 0.698 for a trimester.

**Conclusion:**

As a result of the validity and reliability study, the Turkish version of the Pregnancy Quality of Life Scale can be used as a valid and reliable measurement tool.

## Background

Pregnancy is one of the physiological events in the reproductive period of a woman’s life, which can be experienced by every woman in this period [1]. Pregnancy, which is regarded as a critical developmental period, is a natural process that causes physiological and psychological changes in the life cycle of women and impacts all organs and systems [2]. Different complaints may emerge in each trimester of pregnancy. Conditions such as nausea, vomiting, hemorrhoid, back pain, abdominal discomfort, fatigue, constipation, difficulty sleeping, and gastric problems, which are among the physiological symptoms of pregnancy, can cause distress and adversely impact the physical and mental health of women [3]. Pregnancy-specific physiological changes are the source of psychological changes. Complaints that occur with these changes can adversely influence the quality of life and psychosocial health of pregnant women. Physiological and psychological changes during pregnancy bring the lines of health and disease closer to each other [4–6]. Therefore, pregnancy is a worrisome period for women and can often adversely impact their quality of life [6, 7]. If pregnant women cannot cope with the personal and environmental factors they experience, pregnancy, labor, and motherhood create a crisis environment for them [8]. The inability of pregnant women to adapt adequately to changes experienced during pregnancy may adversely affect the quality of life of expectant mothers [3, 9].

Quality of life covers the person’s health status and personal well-being [10]. The World Health Organization’s Quality of Life (WHOQOL) group defines quality of life as “the patient’s perception of his/her situation in life, both in the context of the cultural structure and values system in which he/she lives and in terms of his/her goals, expectations, standards, and concerns” [11]. Health-related quality of life is a multifactorial term used to describe the physical and psychological state perceived by individuals. It usually consists of the physical and psychic state, pain, fatigue, and social and cognitive function domains. In the healthcare field, quality of life is used to test the consequences of conditions such as allergies, anemia, cancer, obesity, and diabetes. Health-related quality of life is mostly described as “evaluating the effects of the disease and its treatment on the patient, again from the patient’s perspective.” The point that should be emphasized here is that the source used when acquiring the data is the patient himself/herself [12].

Studies on quality of life during pregnancy demonstrate that physiological and psychological changes during pregnancy adversely affect quality of life [4]. For a successful pregnancy, it is necessary to focus on the person’s quality of life during pregnancy. Quality of life during pregnancy is one of the issues that healthcare professionals should focus on and is important to determine prenatal health status.

Midwives have an active role in providing information that will positively affect the quality of life of pregnant women during pregnancy follow-up. In this process, midwives should determine all the needs of pregnant women and help women adapt to pregnancy [3]. They should provide training opportunities for women to adapt to pregnancy and motherhood. Physiological and psychological differences experienced by expectant mothers during pregnancy and their need for social support in this process should be explained to their husbands and families, and counseling on how to help them should be provided [3]. Midwives need some assessment instruments while performing these tasks. Health-related quality of life is a subset of quality of life as a whole. In the literature, health-related quality of life scales are divided into two main headings, general and specific scales [12].

When we look at the standard scales measuring the quality of life during pregnancy in our country, only the Pregnancy Symptoms Inventory and Quality of Life Gravidarum Questionnaire are encountered [13–15]. It has been reported that it is a valid scale to evaluate the complaints during pregnancy and their impacts on quality of life [14]. The Quality of Life Gravidarum (QOL-GRAV) Questionnaire was developed by Vachkova et al. [16] to optimally assess the quality of life of women with normal pregnancies due to the absence of specific tools to measure the general well-being and quality of life of women during pregnancy. In Turkey, instead of focusing on women’s specific problems during pregnancy, there is a need for a tool that can objectively identify them and evaluate their quality of life. The developed Quality of Life Gravidarum Questionnaire has been accepted as a valid and reliable instrument to determine the quality of life of women in Turkish society during pregnancy. It has limitations, such as being applied in a single center and the obtained data is based on pregnant women’s statements [16]. Translating the 9-item QOL-GRAV questionnaire, adapted to different cultures for Turkish women in the antenatal period, into Turkish and determining its validity and reliability will contribute significantly to the national literature. The difference between this scale and other scales is that a single quality of life score is achieved. The objective of this study is to determine the applicability, validity, and reliability of the Turkish version of QOL-GRAV.

## Method

### Research type

The present research is a methodological study.

### Place and time of the study

The study was conducted at Bayburt State Hospital, the only public hospital operating in the city center of Bayburt, between April 2021 and April 2022.

### Study sample

In validity and reliability studies, it is stated that the sample size should be 5-10 times the total number of items [17]. Since the scale’s number of items is 9 in this study, the sample size should be at least 45-90. Since it was thought that studying the scale with a larger group would increase the scale’s measuring power, it was planned to include 9*40=360 pregnant women in the study by taking the sample size as 40 times the scale’s number of items. It was aimed to include a similar number of pregnant women from each trimester, and the sample size of the study consisted of a total of 355 pregnant women, 119 from the first trimester, 110 from the second trimester, and 126 from the third trimester. The target sample size was reached in the study, and the non-probability sampling method was employed to determine the sample size. Our reasons for choosing the non-probability sampling method; the decline in response rates in probability surveys, the high cost of data collection, the increased burden on respondents, the desire for access to real-time Statistics.

### Inclusion and exclusion criteria

#### Inclusion criteria

Pregnant women who could speak and understand Turkish were over the age of 18, were at least primary school graduates, and were healthy or at a low risk were included in the study.

#### Exclusion criteria

Pregnant women who were at risk or high risk and diagnosed with mental illness were not included in the study. Table 1 shows the distribution of pregnant women’s demographic characteristics.

**Table 1:** Distribution of pregnant women according to demographic characteristics

		**1st Trimester (*n* = 119)**		**2nd Trimester(*n* = 110)**		**3rd Trimester (*n* = 126)**	
		**n**	**%**	**n**	**%**	**n**	**%**
**Family Structure**	Nuclear	115	96.6	98	89.1	112	88.9
	Extended	4	3.4	12	10.9	14	11.1
**Educational Status**	Secondary school	37	31.1	24	21.8	39	30.9
	High school	47	39.5	44	40.0	55	43.7
	University	35	29.4	42	38.2	32	25.4
**Occupation**	Housewife	88	73.9	79	71.8	104	82.5
	Civil servant	27	22.7	27	24.5	13	10.3
	Other	4	3.4	4	3.7	9	7.2
**Spouse’s Education**	Secondary school	19	16.0	10	9.1	19	15.1
	High school	56	47.0	46	41.8	50	39.7
	University	44	37.0	54	49.1	57	45.2
**Spouse’s Occupation**	Worker	13	10.9	7	6.4	19	15.1
	Self-employed	47	39.5	43	39.0	34	27.0
	Civil servant	42	35.3	51	46.4	46	36.5
	Other	17	14.3	9	8.2	27	21.4
**Place of Residence**	City	87	73.1	76	69.1	84	66.7
	District	32	26.9	34	30.9	42	33.3
**Social Security**	Available	118	99.2	102	92.7	118	93.7
	Non-available	1	0.8	8	7.3	8	6.3
**Perceived Economic Situation**	Poor	9	7.5	5	4.6	10	7.9
	Medium	86	72.3	70	63.6	78	61.9
	Good	24	20.2	35	31.8	38	30.2
**Smoking**	Yes	11	9.2	12	10.9	7	5.6
	No	108	90.8	98	89.1	119	94.4
**Social Support During Pregnancy**	Yes	84	70.6	82	74.5	105	83.3
	No	35	29.4	28	25.5	21	16.7
		**1st Trimester**		**2nd Trimester**		**3rd Trimester**	
		**n**	**%**	**n**	**%**	**n**	**%**
**The Person from Whom Support Was Received**	Spouse	34	40.5	40	48.8	43	41.0
	Mother/Family	27	32.1	16	19.5	37	35.2
	Multiple choice	23	27.4	26	31.7	25	23.8
**Number of Prenatal Care Received**	1–2 times	67	56.3	15	13.6	87	69.1
	3–4 times	42	35.3	67	60.9	10	7.9
	5–6 times	10	8.4	28	25.5	29	23.0
**Place of Receiving Prenatal Care**	State	104	87.4	83	75.5	102	81.0
	Other (multiple choice)	15	12.6	27	24.5	24	19.0
		**Mean**	**SD**	**Mean**	**SD**	**Mean**	**SD**
Age		28.96	3.69	28.62	4.18	29.41	4.10
BMI		27.06	3.26	27.49	3.42	28.66	3.81
Number of pregnancies		2.25	0.94	2.29	1.03	2.47	1.21
Number of living children		0.93	0.82	0.95	0.86	1.20	1.00
Week of gestation		9.53	1.65	17.94	2.93	35.82	4.27

### Data collection

The researcher collected the data through face-to-face interviews with pregnant women presenting to the gynecology and obstetrics outpatient clinics of Bayburt State Hospital. The “Personal Information Form” (in 1–2 min), the “World Health Organization’s Quality of Life Scale-BREF (WHOQOL-BREF)” (in 3–4 min), the “Complaints During Pregnancy and Their Impacts on Quality of Life Scale (CPIQOLS)” (in 2–3 min), and the “Quality of Life Gravidarum (QOL-GRAV)” questionnaire (in 2–3 min) were used in data collection, and data were collected in 8–10 min.

### Data collection tools

#### Personal information form

This form, prepared by the researcher using the literature on the subject, includes a total of 17 questions regarding the socio-demographic characteristics and obstetric history of pregnant women [18–20].

#### The Quality of Life Gravidarum (QOL-GRAV) questionnaire

The QOL-GRAV (Quality of life gravidarum) questionnaire, developed by Vachkova et al., aims to optimally assess the quality of life of women with normal pregnancies and consists of nine items. Since QOL-GRAV provides a better perspective on the quality of life of pregnant women than a general quality of life scale, it is a scale specific to singleton pregnancy that should be used in clinical and social studies [16]. The responses to the QOL-GRAV questionnaire are evaluated on a five-point Likert scale, and the responses to items 1, 2, 3, 4, 5, and 6 are scored as (1 = Not at all, 2 = A little, 3 = Moderately, 4 = Mostly, and 5 = Completely), and the responses to items 7, 8, and 9 are scored as (1 = Very satisfied, 2 = Satisfied, 3 = Undecided, 4 = Not satisfied, and 5 = Vey dissatisfied). The total score of the scale varies between 9–45 points, and according to the total score of the quality of life scale, points 9–18 are considered as excellent, points 19–27 as very good, points 28–36 as good, and points 37–45 as not very good. Cronbach’s α coefficient of the scale is 0.72, 0.74, and 0.75 according to the first trimester, second trimester, and third trimester, respectively, and it is 0.796 in the study by Mirghafourvand et al. [20], 0.790 in the study by Ishaq et al. [21], and 0.87 in the study by Mazúchová et al. [22].

#### Complaints During Pregnancy and Their Impacts on Quality of Life Scale (CPIQOLS)

The CPIQOLS, developed by Foxcroft et al. [13], aims to determine how often the possible complaints during pregnancy are experienced and how each of them impacts daily life, and it is recommended to be used by healthcare professionals. The scale has 42 items and consists of two sections. The first section evaluates how often the complaints that occur during pregnancy were encountered in the last month; this section is a four-point Likert scale and is coded as “never” (0), “rarely” (1), “sometimes” (2), and “often” (3). If 1–3 is marked for each complaint from the first section, the second section is passed. The second section is a three-point Likert scale that measures how the complaints affect the activities of daily living and is marked as “not limiting at all (0)”, “limiting little (1)”, and “limiting a lot (2)”. The scale has no cut-off point. With an increase in the total score, quality of life decreases. Gür conducted the Turkish validity and reliability study of the scale in 2016. Cronbach’s alpha reliability coefficient of the scale is 0.91 [14]. We used the parallel form of reliability, also known as alternative form reliability, to determine the consistency and equivalence of multiple versions or forms of a measurement instrument intended to measure the same construct.

#### World Health Organization’s Quality of Life Scale-BREF (WHOQOL-BREF)

The World Health Organization developed the comprehensive World Health Organization’s Quality of Life (WHOQOL), which assesses a person’s well-being and allows cross-cultural comparisons [23, 24]. The WHOQOL-BREF is a scale consisting of 26 questions and 4 domains, created by taking one question for 24 sections from the WHOQOL-100 and adding two questions covering general health and quality of life [25]. These four domains are physical, psychological, environmental, and social relationships domains. The WHOQOL-BREF was translated into more than 20 languages, including Turkish. Eser et al. performed the Turkish validity and reliability study of the WHOQOL-BREF. A national question was added to the Turkish validity and reliability study. The participants were asked to answer the questions considering the last 15 days. It is a Likert scale and is based on the logic of scoring one’s quality of life. The scoring of the physical, social relationships, psychological, and environmental domains was calculated using the questions, except for the first two questions, which are quality of life and general health questions. Quality of life also increases with an increase in the domain scores calculated over 0–20 points [26]. The WHOQOLBREF covers four individual domains Physical health, Physiological health, Social relationships, and Environmental health issues. Higher WHOQOL-BREF scale scores indicate a better quality of life [27, 28]. “Cronbach’s α” values calculated for the scale’s internal consistency in the Turkish reliability study were as follows: 0.83 in the physical domain, 0.53 in the social relationships domain, 0.66 in the psychological domain, 0.73 in the environment domain, and 0.73 in the national environment domain [26]. We used the parallel form of reliability, also known as alternative form reliability, to determine the consistency and equivalence of multiple versions or forms of a measurement instrument intended to measure the same construct.

### Validity and reliability of the Turkish-English adaptation of the Quality of Life Gravidarum (QOL-GRAV) questionnaire

In the adaptation study of the QOL-GRAV questionnaire into Turkish, first, language and content validity were examined, and then the construct validity and internal consistency coefficients were analyzed.

#### Language validity

First, the researcher and two academicians translated the QOL-GRAV questionnaire from English to Turkish. The scale items translated into Turkish were reviewed by the researcher and the advisor, and a single form was created. Afterward, two domain experts who knew both languages back-translated the scale translated into Turkish. The original version of the scale was compared with its Turkish translation, and it was stated that there was no change in the scale items in any sense. Thus, the Turkish translation of the scale was completed. After the translation process was completed and the necessary permissions were obtained, the scale was applied to a pilot group of 20 pregnant women. These 20 pregnant women were not included in the study. After applying the scale to the pilot group, it was found that the items were understandable. Afterward, expert opinion was sought for content validity.

#### Content validity

After the translation of the scale, the English and Turkish forms of the scale were sent via e-mail to 10 experts in the field for content validity. The experts were asked for their opinions on the plainness and clarity of the statements in the scale and the evaluation of the conformity of the statements to our culture. They were also asked to interpret each item in the scale separately in the range of 1–4 points as 4: “Absolutely appropriate,” 3: “Appropriate,” 2: “Slightly appropriate,” and 1: “Inappropriate.” Expert opinions were obtained using the Davis method. According to the opinions, the statements found to be appropriate by the experts were accepted in the same way, and the statements that should be corrected or were found inappropriate were re-examined, and corrections were made. After this evaluation, the number of experts who said that the items were appropriate and appropriate was divided by the total number of experts, and the content validity index (CVI) was found. A CVI value higher than 0.80 indicates the conformity of the statement in terms of content validity [29]. The scale was finalized with expert suggestions.

#### Construct validity

Factor analysis was applied to determine the construct validity of the Quality of Life Gravidarum Questionnaire. Before the factor analysis, KMO and Bartlett’s tests were conducted to determine the sample adequacy and the suitability of the data for factor analysis.

#### Internal consistency

The scale’s internal consistency was examined by Cronbach’s alpha coefficient and item-total score correlations. Cronbach’s alpha coefficient must be at least 0.60, and the item-total correlations must be at least 0.20 in each statement [30].

### Data evaluation

The Statistical Package for Social Science version 22.0 (SPSS Inc; Chicago, IL, USA) and LISREL 8.80 package programs were used in data evaluation. Descriptive statistics were employed in the analysis of descriptive data. The Davis technique and Content Validity Index (CVI) were used for content/scope validity in data analysis; the Kaiser-Mayer-Olkin (KMO) Index, principal component analysis, and Bartlett’s test (BTS) were used for sample size and suitability of the data set for factor analysis; exploratory and confirmatory factor analyses were used for construct validity; translation/back-translation and expert group review were used for language validity; Cronbach’s alpha coefficient, parallel forms reliability, and item-total correlation were used for internal consistency.

### Ethical considerations of the study

Permission was obtained by e-mail from Eva Vachková, who developed the scale, for the adaptation of the Quality of Life Gravidarum Questionnaire to Turkish and the use of its Turkish version. Approval from the Clinical Research Ethics Committee of Atatürk University Faculty of Medicine (Date: 04.03.2021, Decision No: 01/22) and written permission from Bayburt Provincial Health Directorate were obtained to conduct the study. Moreover, verbal consent was obtained from the pregnant women who agreed to participate in the study voluntarily. Consent was obtained from all participants. The Declaration of Helsinki rules were followed at every stage of the research.

### Limitations and generalizability of the study

This study has some limitations. The reliability of the data in the data collection tools is limited to the accuracy of the information given by the pregnant women. The fact that the study was performed in Bayburt State Hospital affiliated with the Bayburt Provincial Health Directorate and that the sample was created using the improbable sampling method are the other limitations of the study. Data was collected from a single center to collect more data from the institution where the researcher works. The study results can only be generalized to the group that presented to Bayburt State Hospital and resembled the pregnant women within the scope of the study.

### Funding

No funding was received for this study.

## Results

Language validity, content validity, and construct validity were measured to ensure the validity of the Quality of Life Gravidarum (QOL-GRAV) Questionnaire, whereas internal consistency was measured to ensure its reliability.

### Validity analyses

#### Language validity

The validity and reliability study of the Quality of Life Gravidarum Questionnaire was first translated from English into Turkish by the researcher and two academicians. The scale items translated into Turkish were reviewed by the researcher and the advisor, and a single form was created. Then two linguists who had not seen the English version of the scale before and knew both languages back-translated the scale translated into Turkish from Turkish to English. Subsequently, the forward and backward translations were compared in the panel including the researcher and the linguist who knew both languages, and it was stated that there was no change in the scale items in any sense. Thus, the Turkish translation of the scale was completed. After the translation process was completed and the necessary permissions were obtained, the scale was applied to a pilot group of 20 pregnant women. These 20 pregnant women were not included in the study. After applying the scale to the pilot group, it was determined that the items were understandable.

#### Content validity

After the language validity of the Quality of Life Gravidarum Questionnaire was ensured, the opinions of 10 experts were obtained to ensure the content validity. The Davis technique was employed in evaluating expert opinions. The experts were asked to rate each item on the scale with scores ranging from “1” to “4” as “4: Appropriate,” “3: Appropriate,” “2: Slightly appropriate,” and “1: Inappropriate.” The expert opinions were evaluated using the Davis method.

#### Construct validity

The construct validity method was employed to evaluate how accurately the scale measures the quality of life during pregnancy. Factor analysis was applied to determine the construct validity of the Quality of Life Gravidarum Questionnaire. Before the factor analysis, Kaiser–Meyer–Olkin (KMO) and Bartlett’s tests were conducted to determine sample adequacy and suitability of the data for factor analysis. The KMO measure between 0.90–1.00 is accepted as marvelous, between 0.80–0.89 as meritorious, between 0.70–0.79 as average, between 0.60–0.69 as mediocre, between 0.50–0.59 as terrible, and below 0.50 as unacceptable [31, 32]. Small KMO values indicate that it is not a good idea to conduct a principal component analysis of the variables [33].

The KMO value of QOL-GRAV in the first trimester was 0.615, the KMO value in the second trimester was 0.726, and the KMO value in the third trimester was 0.691. Bartlett’s test value was (× 2 = 224.558, *p* = 0.000) for the first trimester, (× 2 = 283.439, *p* = 0.000) for the second trimester, and (× 2 = 289.599, *p* = 0.000) for the third trimester. In these results, it was concluded that the data were related to each other and the sample size was sufficient at the “very good” level to perform the factor analysis.

#### Exploratory factor analysis

After KMO and Bartlett’s tests, the principal component analysis method was employed to examine the scale’s factor structure. The main purposes of the factor analysis are to reduce the number of variables and show new constructs by making use of the relationship between the variables.

In the principal component analysis, to be able to say that an item measures a construct or factor well, the value of this factor loading must be 0.30 or above [33] because, with an increase in the factor weight, the power of that variable sentence to explain the related factor will increase to the same extent, and this will increase the reliability of the factor. Therefore, the 0.30 criterion was taken into account in determining the factor weight in this study. As a result of the principal component analysis, a single factor structure with an eigenvalue above 1 was revealed in all three trimesters and total, similar to the original structure for the nine items taken as a basis for the analysis.

As seen in Table 2, the contribution of a single factor to the total variance in the Quality of Life Gravidarum Questionnaire is 30.934% for the first trimester, 32.160% for the second trimester, and 31.279% for the third trimester. The factor loadings of all items in the scale are between 0.32 and 0.72. The explained variance is 30.934% in the first trimester, 32.160% in the second trimester, and 31.279% in the third trimester. The range of the factor loadings is 0.349–0.724 for the first trimester, 0.349–0.742 for the second trimester, and 0.327–0.728 for the third trimester. At this stage, no statement was removed from the scale, and it was approved as a single factor in the Turkish language. Büyüköztürk states that the variance explained in one-dimensional structures can be reduced to 30%. It has been stated in the literature that sample size may affect data groups where the common variance is low and the factors are not very stable [31, 32]. According to Maccallum et al. [32], large samples may be needed in cases where the common variance value is low.

**Table 2:** Factor pattern of the statements in the quality of life gravidarum questionnaire

Items	**Factor Loading Values**		
	**1st Trimester**	**2nd Trimester**	**3rd Trimester**
	**Factor 1**	**Factor 1**	**Factor 1**
1	0.724	0.478	0.681
2	0.597	0.581	0.728
3	0.367	0.486	0.582
4	0.642	0.701	0.654
5	0.448	0.742	0.516
6	0.395	0.625	0.389
7	0.421	0.580	0.622
8	0.349	0.449	0.327
9	0.500	0.349	0.378
**Total Variance Explained (%)**	30.934	32.160	31.279

#### Confirmatory factor analysis

After the exploratory factor analysis, confirmatory factor analysis was conducted to test the accuracy of the determined single-factor structure and the suitability of the model for exploratory factor analysis using the LISREL program. After the confirmatory factor analysis, χ2/sd, GFI, AGFI, CFI, RMSEA, and SRMR were examined to assess whether the model’s structure was compatible with the data.

The value formed by proportioning χ2 to the degree of freedom (sd) must be 2 or less than 2. A value of 5 and lower is an acceptable value [34, 35]. GFI means the goodness of fit index. The GFI value varies between 0 and 1. The GFI value exceeding 0.90 is considered to indicate a good model. AGFI is an index used to eliminate the deficiency of the GFI test in a high sample size. Its value varies between 0–1 and must be above 0.90. CFI gives the difference between the established model from the absence model, assuming that there is no relationship between the variables. It is a model predicting that there is no relationship between the variables. Its value varies between 0–1. RMSEA is a measure of approximate fit in the population. Its value changes between 0–1. As the SRMR value approaches zero, it is understood that the tested model shows better goodness of fit [33, 34, 36, 37]. In the literature, it is reported that the RMSEA and SRMR values should be lower than 0.08 and the GFI, AGFI, and CFI values should be above 0.9 [35].

Variables of the model, t-values, factor loadings, unexplained variance, and some fit index values can be observed in an abbreviated form by drawing the PATH diagram. After the PATH diagram is drawn, first, the t-values of the statements are reviewed. If the table t-value exceeds 1.96, it is significant at the 0.05 level; if it exceeds 2.56, it is significant at the 0.001 level. Insignificant ones should be removed from the scale [34]. During the factor analysis, the factor loadings of the scale statements should not be lower than 0.30. A value of 71 and above is accepted as excellent, 0.63 as very good, 0.55 as good, 0.45 as acceptable, and 0.32 as poor [34, 35].

#### Confirmatory factor analysis results according to the first trimester

**Fig. 1:**
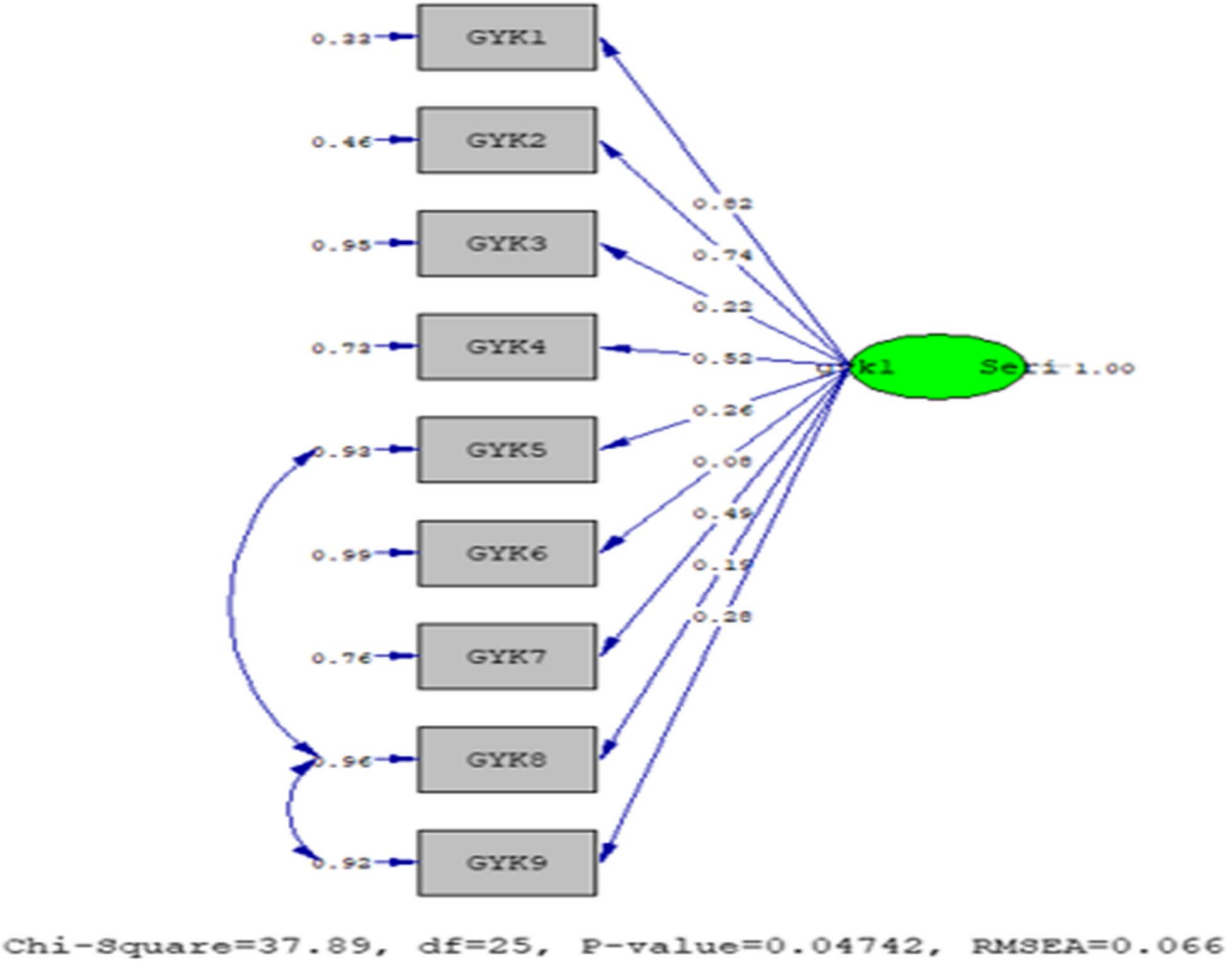
The PATH diagram of the statements for the quality of life Gravidarum questionnaire according to the first trimester

As a result of the confirmatory factor analysis conducted for the statements of the Quality of Life Gravidarum Questionnaire, the normal and acceptable fit index values obtained are presented in Table 3, and the PATH diagram is shown in Fig. 1. According to the fit index values in Table 3, the scale statements were accepted as appropriate [23].

**Table 3:** The normal and acceptable fit ındex values obtained for the quality of life gravidarum questionnaire in the first trimester

Fit Indices	1st Trimester	2nd Trimester	3rd Trimester
**χ**^**2**^**/SD**	1.52	1.63	1.99
**GFI**	0.94	0.92	0.91
**AGFI**	0.89	0.85	0.83
**CFI**	0.99	0.96	0.98
**RMSEA**	0.027	0.011	0.045
**SRMR**	0.066	0.076	0.081

The fit index values obtained for the Quality of Life Gravidarum Questionnaire in the first trimester are as follows; χ2/sd = 1.52, GFI = 0.94, AGFI = 0.89, CFI = 0.99, RMSEA = 0.027, and SRMR = 0.066. The fit index values obtained for the Quality of Life Gravidarum Questionnaire in the second trimester are presented below; χ2/sd = 1.63, GFI = 0.92, AGFI = 0.85, CFI = 0.96, RMSEA = 0.011, and SRMR = 0.076. The following fit index values were obtained for the Quality of Life Gravidarum Questionnaire in the third trimester; χ2/sd = 1.63, GFI = 0.92, AGFI = 0.85, CFI = 0.96, RMSEA = 0.011, and SRMR = 0.076. According to the results of the fit index values, the scale statements were accepted as appropriate [36].

The model was approved in its original form, as shown in Fig. 1 The factor loadings of the model vary between 0.08 and 0.82, and the t-value for all items is above 1.96. No modifications were applied to improve the model.

**Fig. 2:**
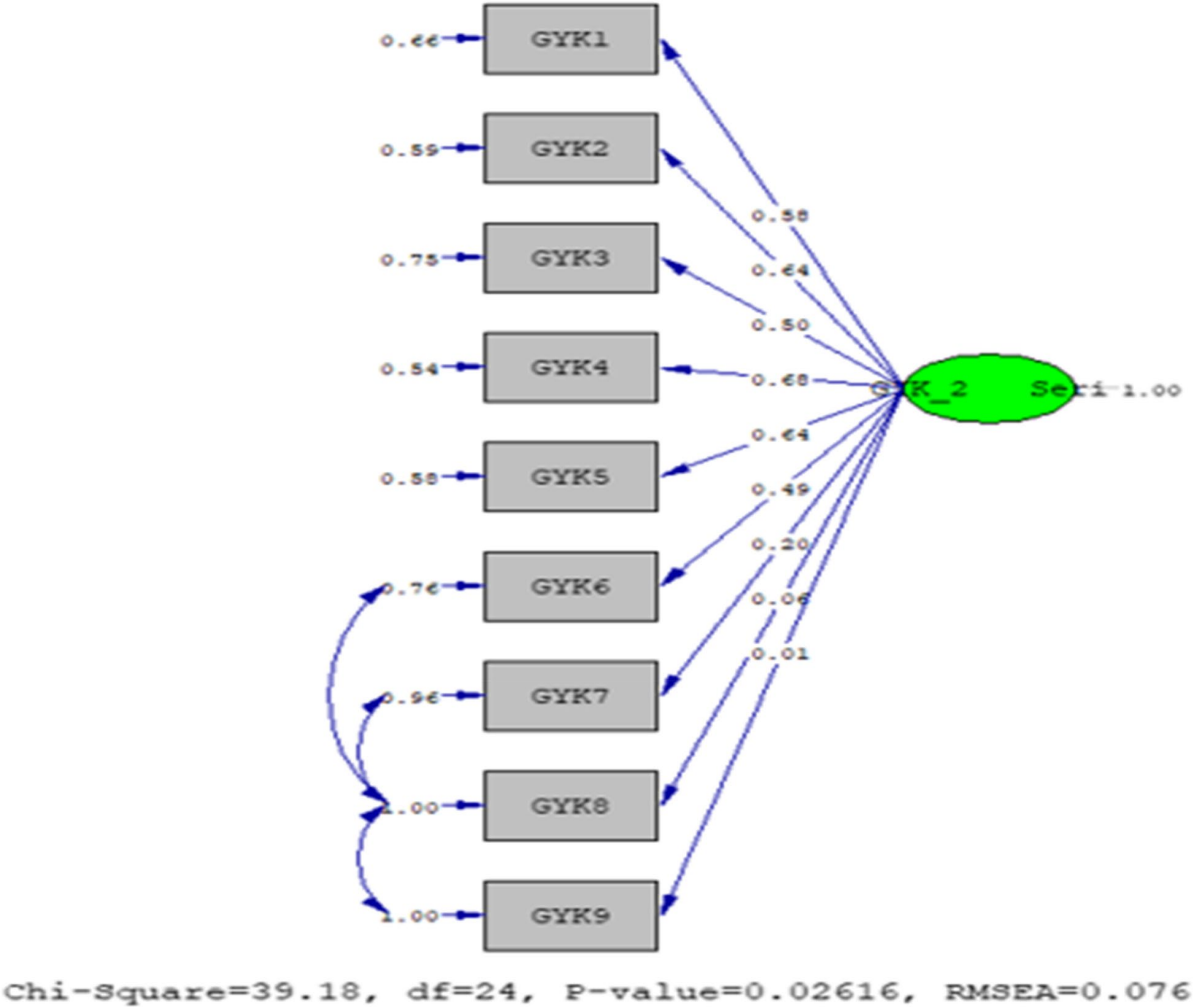
The PATH diagram of the statements for the quality of life Gravidarum questionnaire according to the second trimester

The model was approved in its original form, as shown in Fig. 2 The factor loadings of the model vary between 0.01 and 0.68, and the t-value for all items is above 1.96. No modifications were applied to improve the model.

**Fig. 3:**
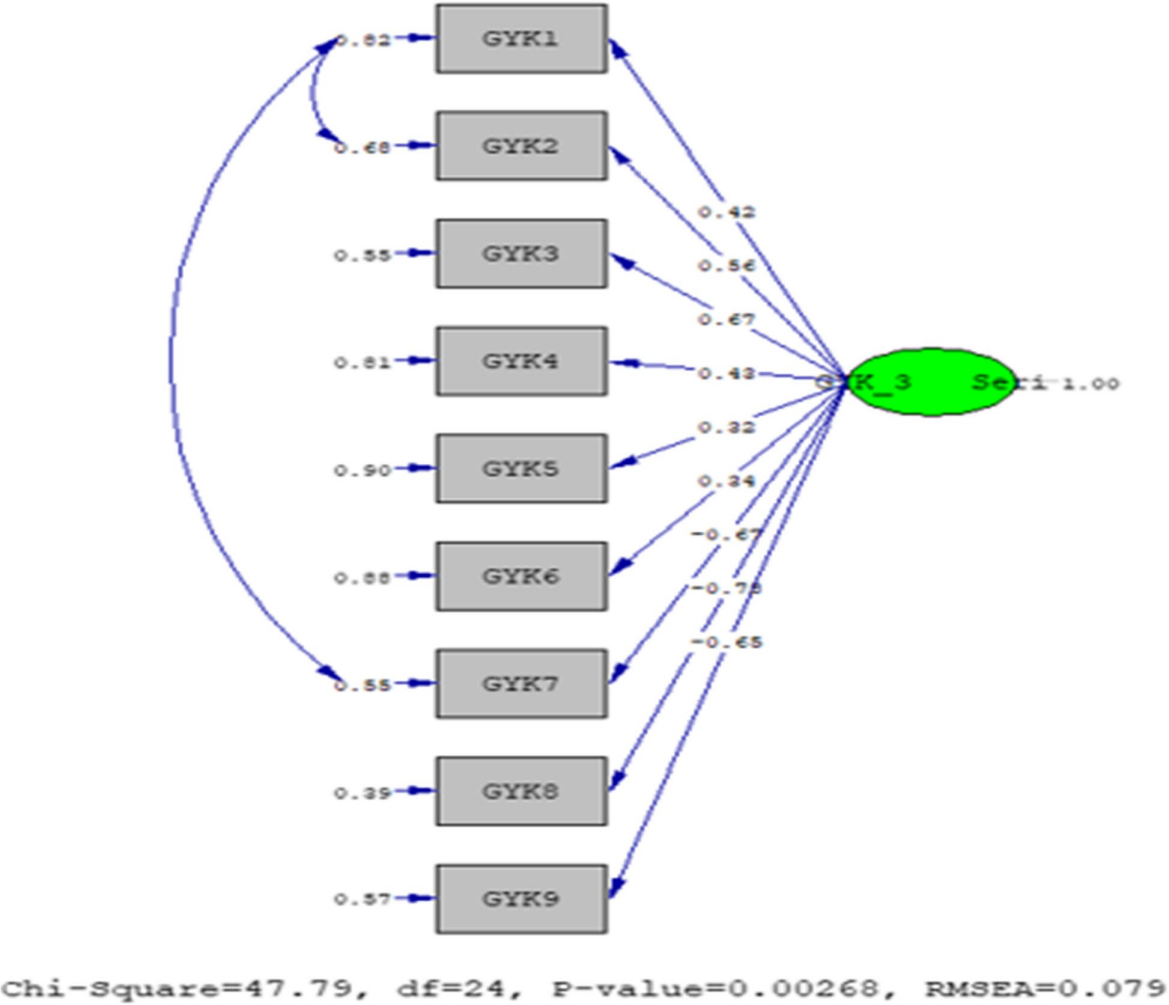
The PATH diagram of the statements for the quality of life Gravidarum questionnaire according to the third trimester

The model was approved in its original form, as shown in Fig. 3 The factor loadings of the model vary between 0.32 and 0.78, and the t-value for all items is above 1.96. No modifications were applied to improve the model.

Upon comparing the fit indices of the Quality of Life Gravidarum Questionnaire with the normal and acceptable fit criteria given in Table 5 in all pregnant women in the 1st-2nd and 3rd trimesters, it was observed that the single-factor structure of the model consisting of 9 items was confirmed as a model.

### Reliability analyses

#### Internal consistency

In the reliability analysis of the Quality of Life Gravidarum Questionnaire, item analysis based on Cronbach’s α reliability coefficient and item-total correlation and parallel forms reliability analysis were carried out to measure internal consistency. Cronbach’s α reliability coefficient must be at least 0.60, and the item-total correlations must be at least 0.20 in each statement [30].

#### Item analysis based on Cronbach’s α reliability coefficient and ıtem-total correlation of the statements for quality of life gravidarum in the first trimester

Cronbach’s α reliability coefficient of the statements in the Quality of Life Gravidarum Questionnaire is α = 0.628 in the first trimester, 0.727 in the second trimester, and 0.698 in the third trimester (Table 5). According to Cronbach’s α values ​​of the scale, which are above 0.60 in all trimesters, the scale is quite reliable and has internal consistency. This means that the items in the scale consist of items measuring the items of the same feature.

The item-total correlations of the statements in the scale are 0.200–0.484 in the first trimester, 0.256–0.566 in the second trimester, and 0.227–0.503 in the third trimester. Accordingly, the item-total correlation values ​​are positive, and the lowest value is 0.200 (Table 4). Thus, it was concluded that all items on the scale moved in the same direction as the overall scale, and no item was removed from the scale. Hence, the scale has internal consistency.

**Table 4:** Item-total correlations and Cronbach’s α reliability coefficient of the statements in the quality of life gravidarum questionnaire in pregnant women by trimesters

	Item No	Mean	SD	Item-total correlation	Cronbach’s α if the item is deleted
**1st Trimester**	1	3.02	0.85	0.484	0.549
	2	2.78	0.73	0.361	0.587
	3	2.29	0.78	0.200	0.626
	4	3.21	0.76	0.426	0.570
	5	3.08	0.87	0.245	0.618
	6	2.95	0.71	0.229	0.618
	7	4.32	0.69	0.255	0.612
	8	4.03	0.67	0.228	0.617
	9	4.13	0.74	0.352	0.589
	Cronbach’s α reliability coefficient		0.628		
	Mean ± SD		29.79 ± 3.35		
**2nd Trimester**	1	2.74	0.75	0.290	0.721
	2	2.53	0.74	0.395	0.704
	3	2.25	0.83	0.316	0.718
	4	2.95	0.84	0.530	0.677
	5	3.05	0.86	0.566	0.669
	6	2.69	0.70	0.461	0.693
	7	4.24	0.79	0.461	0.691
	8	3.93	0.83	0.335	0.715
	9	4.10	0.70	0.256	0.725
	Cronbach’s α reliability coefficient		0.727		
	Mean ± SD		28.55 ± 3.66		
**3rd Trimester**	1	2.94	0.70	0.477	0.658
	2	2.70	0.73	0.503	0.653
	3	2.43	0.95	0.347	0.678
	4	2.90	0.93	0.479	0.650
	5	3.06	1.01	0.343	0.680
	6	2.85	0.85	0.227	0.699
	7	4.15	0.93	0.499	0.646
	8	3.75	1.00	0.253	0.699
	9	3.98	0.86	0.300	0.686
	Cronbach’s α reliability coefficient		0.698		
	Mean ± SD		29.44 ± 3.35		

The item-total correlation is the correlation between a scored item and the total test score. In this study, psychometric analysis was used to identify assessment items that fail to indicate the underlying psychological trait so that they can be removed or revised. The mean score of the Quality of Life Gravidarum Questionnaire is 29.79 ± 3.35 in the first trimester, 28.55 ± 3.66 in the second trimester, and 29.44 ± 3.35 in the third trimester (Table 5).

**Table 5:** Parallel forms reliability results

Trimester			WHOQOL				CPIQOLS
			**Physical Domain**	**Psychological Domain**	**Social Domain**	**Environment Domain**	
1	**QOL-GRAV**	r	0.705	0.708	0.709	0.715	0.890
		p^*^	**0.000**	**0.000**	**0.000**	**0.000**	**0.000**
2	**QOL-GRAV**	R	0.725	0.702	0.722	0.715	0.881
		p^*^	**0.000**	**0.000**	**0.000**	**0.000**	**0.000**
3	**QOL-GRAV**	R	0.719	0.705	0.704	0.701	0.893
		p^*^	**0.000**	**0.000**	**0.000**	**0.000**	**0.000**

^*^Spearman’s Rho

In the validity and reliability study of QOL-GRAV, the WHOQOL and the Complaints During Pregnancy and Their Impacts on Quality of Life Scale were used as a parallel form. Table 5 shows the parallel forms reliability results. All values ​​were determined to be acceptable.

Parallel forms reliability is a measure of reliability obtained by administering different scales of an assessment tool (both versions must contain items that probe the same construct, skill, knowledge base, etc.) to the same group of individuals. The same group of respondents answers both sets, and you calculate the correlation between the results. A high correlation between the two indicates high parallel forms reliability. There is a statistically positive high correlation between the Quality of Life Gravidarum in the first trimester and the Physical domain of the WHOQOL (*r* = 0.705; *p* = 0.000). A statistically positive high correlation is revealed between the Quality of Life Gravidarum in the first trimester and the Psychological domain of the WHOQOL (*r* = 0.708; *p* = 0.000). A statistically positive high correlation is found between the Quality of Life Gravidarum in the first trimester and the Social domain of the WHOQOL (*r* = 0.709; *p* = 0.000). There is a statistically positive high correlation between the Quality of Life Gravidarum in the first trimester and the Environment domain of the WHOQOL (*r* = 0.715; *p* = 0.000). A statistically positive high correlation is determined between the Quality of Life Gravidarum in the second trimester and the Physical domain of the WHOQOL (*r* = 0.725; *p* = 0.000). A statistically positive high correlation is revealed between the Quality of Life Gravidarum in the second trimester and the Psychological domain of the WHOQOL (*r* = 0.702; *p* = 0.000). There is a statistically positive high correlation between the Quality of Life Gravidarum in the second trimester and the Social domain of the WHOQOL (*r* = 0.722; *p* = 0.000). A statistically positive high correlation is found between the Quality of Life Gravidarum in the second trimester and the Environment domain of the WHOQOL (*r* = 0.715; *p* = 0.000). There is a statistically positive high correlation between the Quality of Life Gravidarum in the third trimester and the Physical domain of the WHOQOL (*r* = 0.719; *p* = 0.000). A statistically positive high correlation is determined between the Quality of Life Gravidarum in the third trimester and the Psychological domain of the WHOQOL (*r* = 0.705; *p* = 0.000). A statistically positive high correlation is revealed between the Quality of Life Gravidarum in the third trimester and the Social domain of the WHOQOL (*r* = 0.704; *p* = 0.000). There is a statistically positive high correlation between the Quality of Life Gravidarum in the third trimester and the Environment domain of the WHOQOL (*r* = 0.701; *p* = 0.000). With an increase in the WHOQOL score, the score of the Quality of Life Gravidarum also increases (*p* < 0.05).

A statistically negative and high correlation was obtained between the Quality of Life Gravidarum Questionnaire in the first trimester and the CPIQOLS (*r* = -0.890; *p* = 0.000). A statistically negative and high correlation was found between the Quality of Life Gravidarum Questionnaire in the second trimester and the CPIQOLS (*r* = -0.881; *p* = 0.000). A statistically negative and high correlation was obtained between the Quality of Life Gravidarum Questionnaire in the third trimester and the CPIQOLS (*r* = -0.893; *p* = 0.000). Since Spearman’s rho coefficient, which is the parallel forms reliability coefficient, is higher than 0.70, the expected level of correlation is observed between the forms. In the study, the scales show high correlation and high parallel form reliability.

## Discussion

In this section, the results of the validity and reliability analyses for the Turkish version of the Quality of Life Gravidarum Questionnaire are discussed.

In the scale’s adaptation, first, the scale should be translated. In the language adaptation of the Quality of Life Gravidarum Questionnaire, the translation-back translation technique, the most preferred method in the world, was used to preserve the cultural equality of the scale, despite its requiring time and cost. In the validity and reliability study of the Quality of Life Gravidarum Questionnaire, the researcher and two academicians translated it from English to Turkish. The scale items translated into Turkish were checked again by the researcher and the advisor, and a single form was created. Afterward, two experts who knew both languages back-translated the scale translated into Turkish. Upon comparing the Turkish version with the original version of the scale, it was stated that there was no change in the scale items in any sense. Then the scale was applied to a pilot group of 20 pregnant women. After applying the scale to the pilot group, it was revealed that the items were understandable. Thus, the Turkish translation of the scale was completed, and the scale was finalized. As a result of these studies, it can be said that the Turkish version of the Quality of Life Gravidarum Questionnaire is a suitable assessment instrument in terms of language validity.

The Davis technique, based on the agreement of the majority of the experts, was employed for content validity. After the translation was completed, the opinions of 10 experts were obtained to ensure content validity. The experts were asked to interpret each item in the scale separately in the range of 1–4 points as 4: “Absolutely appropriate”. 3: “Appropriate”. 2: “Slightly appropriate”. 1: “Inappropriate.” It is thought that the number of experts should be between 3 and 20 people when calculating the content validity index [29]. In this respect, obtaining the opinion of 10 experts about the scale is in line with the literature.

It was indicated that the CVI score should be 0.80 and above in the content validity using the Davis method [29]. In the present study, the CVI score was determined as 1.00 for all statements in the scale. Thus, it can be concluded that the scale is sufficient in terms of content validity.

Factor analysis is the best method of observing statistically the construct validity [38]. Before the factor analysis of the Quality of Life Gravidarum Questionnaire, KMO, and Bartlett’s tests were applied to determine the sample adequacy and whether the data were suitable for factor analysis. In our study, the KMO value was 0.615 in the first trimester, 0.726 in the second trimester, and 0.691 in the third trimester. These results show the adequacy and suitability of the sample for factor analysis. Bartlett’s test gives the X2 (chi-square) value. The significance value is checked, as in the X2 test. In the current study, it resulted as (× 2 = 224.558, *p* = 0.000) for the first trimester, (× 2 = 283.439, *p* = 0.000) for the second trimester, and (× 2 = 289.599, *p* = 0.000) for the third trimester. These results indicate the adequacy and suitability of the sample for factor analysis.

The validity criteria of the Quality of Life Gravidarum Questionnaire were evaluated by examining the factor structure first, and exploratory factor analysis was applied after KMO and Bartlett’s tests. For the Quality of Life Gravidarum Questionnaire, a single-factor structure was obtained in all three trimesters, similar to the original structure. This study determined that the single-factor structure of the scale was valid for our country since the factor loadings of all items in the scale were between 0.32 and 0.72 and above 0.30. These values are similar to the study of Vachkova et all and Özer [15, 16].

The single-factor total variance of the items in the Quality of Life Gravidarum Questionnaire is 30.934% in the 1st trimester, 32.160% in the 2nd trimester, and 31.279% in the 3rd trimester. Thus, it was revealed that the factor loadings and explained variance were sufficient. Büyüköztürk states that the variance explained in one-dimensional structures can be reduced to 30%. It has been stated in the literature that sample size may affect data groups where the common variance is low and the factors are not very stable [31, 32]. According to Maccallum et al. [32], large samples may be needed in cases where the common variance value is low.

In our study, after the exploratory factor analysis, a confirmatory factor analysis was conducted to obtain clearer results. Many indices were used to review the suitability of the model of the Quality of Life Gravidarum Questionnaire. For the first trimester, the × 2/SD value was determined as 1.52, GFI as 0.94, AGFI as 0.89, CFI as 0.99, RMSEA as 0.027, and SRMR as 0.066. For the second trimester, the × 2 /SD value was found as 1.63, GFI as 0.92, AGFI as 0.85, CFI as 0.96, RMSEA as 0.011, and SRMR as 0.076. For the third trimester, the × 2 /SD value was revealed to be 1.99, GFI 0.91, AGFI 0.83, CFI 0.98, RMSEA 0.045, and SRMR 0.081. In this study using fit indices to review the suitability of the model of the Quality of Life Gravidarum Questionnaire, it is a positive result that the single-factor structure of the Quality of Life Gravidarum Questionnaire was supported by CFA. As a result, the Quality of Life Gravidarum Questionnaire was approved to have a single-factor structure in the Turkish language, and the scale’s construct validity was ensured.

In scale adaptation studies, the scale adapted to the target language should also be tested in terms of reliability. Cronbach’s α coefficient, which is used to measure the internal consistency of a Likert scale, takes a value in the range of 0.00–1.00, and the reliability increases as the value approaches 1.00. In the adaptation study of the Quality of Life Gravidarum Questionnaire, information about its reliability was obtained by examining the internal consistency coefficient. When evaluated according to Cronbach’s α internal consistency coefficient criterion, the Turkish version of the scale has high internal consistency with Cronbach’s α value of 0.72 for the first trimester, 0.74 for the second trimester, and 0.75 for the third trimester, and original version has high internal consistency with Cronbach’s α value of 0.87 for the first trimester, 0.67 for the second trimester, and 0.79 for the third trimester, similar with Cronbach’s α value of Vachkova et al. [16], Mazúchová et al. [22] and Mirghafourvand et al. [20].

Another method used to assess internal consistency is the item-total score correlation. It shows the connection between the scores obtained from the statements in the assessment instrument and the total score. The positive and high value of the item-total correlation indicates that the statements in the assessment instrument exemplify similar situations and the internal consistency of the scale is high [37]. In the literature, it is reported that “the item-total score correlation coefficient value between 0.00–0.19 indicates little/no distinction, the value between 0.20–0.39 is accepted as moderate distinction, the value between 0.40–1.00 means good distinction” [33]. The Item-Total Score Correlation coefficients, showing the connection between the scores obtained from the statements in the assessment instrument and the total score and evaluating internal consistency, varied between 0.20–0.56 in the 1st-2nd and 3rd trimesters in this study, while they were distributed between 0.29–0.69 in the 1st-2nd and 3rd trimesters in the original scale, which is an important result in terms of the similarity of the obtained data [16].

In parallel forms reliability method, according to Spearman’s rho coefficient, there is a positive and moderate correlation (*p* < 0.05) between the Quality of Life Gravidarum Questionnaire and the WHOQOL with the Physical, Psychological, Social, and Environment Domains and a negative and high correlation (*p* < 0.05) with the Complaints During Pregnancy and Their Impacts on Quality of Life Scale. It is a desired positive result that as the mean score of the WHOQOL increases, the mean score of the Quality of Life Gravidarum Questionnaire increases, and as the score of the Complaints During Pregnancy and Their Impacts on Quality of Life Scale increases, the score of the Quality of Life Gravidarum Questionnaire decreases.

## Conclusion and recommendations

This study sought an answer to the question, “Is the Quality of Life Gravidarum (QOL-GRAV) Questionnaire a valid and reliable assessment instrument in Turkish?”. To this end, the scale was evaluated in terms of language validity, content validity, construct validity, and reliability.

The QOL-GRAV, developed by Vachkova et al. [16], aims to optimally assess the quality of life of women with normal pregnancies and consists of nine items. Since the QOL-GRAV questionnaire provides a better perspective on the quality of life of pregnant women than a general quality of life scale, it is a scale specific to singleton pregnancy that should be used in clinical and social studies. The responses to the QOL-GRAV questionnaire are evaluated on a five-point Likert scale, and the responses to items 1, 2, 3, 4, 5, and 6 are scored as (1 = Not at all, 2 = A little, 3 = Moderately, 4 = Mostly, and 5 = Completely), and the responses to items 7, 8, and 9 are scored as (1 = Very satisfied, 2 = Satisfied, 3 = Undecided, 4 = Not satisfied, and 5 = Vey dissatisfied). The total score of the scale varies between 9–45 points, and according to the total score of the quality of life scale, points 9–18 are considered as excellent, points 19–27 as very good, points 28–36 as good, and points 37–45 as not very good. Cronbach’s α coefficient of the scale is 0.72 in the first trimester, 0.74 in the second trimester, and 0.75 in the third trimester.

As a result of the validity and reliability study;

- The language validity of the Quality of Life Gravidarum Questionnaire was analyzed by the translation-back-translation method, and the content validity was ensured with expert opinions.
- The results of KMO and Bartlett’s tests were found to be sufficient (KMO = 0.615, Bartlett’s test (x2 = 224.558, *p* = 0.000) in the first trimester, KMO = 0.726, Bartlett’s test (x2 = 283.439, *p* = 0.000) in the second trimester, KMO = 0.691, Bartlett’s test (x2 = 289.599, *p* = 0.000) in the third trimester).
- According to the exploratory factor analysis results, the factor loadings of all items in the scale (0.32–0.72) and explained variance (30.934% in the first trimester, 32.160% in the second trimester, and 31.279% in the third trimester) were revealed to be sufficient.
- The exploratory factor analysis of the scale determined that it showed a single-factor structure.
- Cronbach’s α coefficient (0.628 in the first trimester, 0.727 in the second trimester, and 0.698 in the third trimester) was found to be at a sufficient level.
- It was revealed that the item-total correlations of all items were at a sufficient level. As a result, the Quality of Life Gravidarum Questionnaire was determined to be a valid and reliable instrument in Turkish society.

In line with these results, the following recommendations can be made:

The Quality of Life Gravidarum Questionnaire;

- Should be used in studies on women’s general well-being and quality of life during pregnancy,
- Should be used by midwives and other healthcare professionals providing service to pregnant women as a tool for pregnant women’s quality of life levels during pregnancy,
- The quality of life during pregnancy of women with different socioeconomic, sociodemographic, and sociocultural characteristics should be evaluated through the QOL-GRAV.
- Implementing the Quality of Life Gravidarum Questionnaire in different languages and cultures in future research will contribute to improving the scale’s validity and reliability.
- The Quality of Life Gravidarum Questionnaire can be applied to Turkish women in all periods of pregnancy.
- The subjective perceptions of pregnant women regarding their health-related quality of life are a fundamental measure of the quality and effectiveness of maternal and child health interventions. It is important in terms of determining and evaluating the quality of life levels of pregnant women and being an important resource provider for health authorities to establish health policies.
- This scale will measure the ability of pregnant women to perform the normal tasks of life.

## Data Availability

No datasets were generated or analysed during the current study.
